# Chronic Kidney Disease Cohort Studies: A Guide to Metabolome Analyses

**DOI:** 10.3390/metabo11070460

**Published:** 2021-07-16

**Authors:** Ulla T. Schultheiss, Robin Kosch, Fruzsina Kotsis, Michael Altenbuchinger, Helena U. Zacharias

**Affiliations:** 1 Institute of Genetic Epidemiology, Faculty of Medicine and Medical Center, University of Freiburg, 79106 Freiburg, Germany; ulla.schultheiss@uniklinik-freiburg.de (U.T.S.); fruzsina.kotsis@uniklinik-freiburg.de (F.K.); 2 Department of Medicine IV—Nephrology and Primary Care, Faculty of Medicine and Medical Center, University of Freiburg, 79106 Freiburg, Germany; 3 Computational Biology, University of Hohenheim, 70599 Stuttgart, Germany; robin.kosch@uni-hohenheim.de; 4 Institute of Medical Bioinformatics, University Medical Center Göttingen, 37077 Göttingen, Germany; michael.altenbuchinger@med.uni-goettingen.de; 5 Department of Internal Medicine I, University Medical Center Schleswig-Holstein, Campus Kiel, 24105 Kiel, Germany; 6 Institute of Clinical Molecular Biology, Kiel University and University Medical Center Schleswig-Holstein, Campus Kiel, 24105 Kiel, Germany

**Keywords:** metabolomics study design, nephrology, chronic kidney disease, human cohort studies, epidemiology, kidney disease etiologies

## Abstract

Kidney diseases still pose one of the biggest challenges for global health, and their heterogeneity and often high comorbidity load seriously hinders the unraveling of their underlying pathomechanisms and the delivery of optimal patient care. Metabolomics, the quantitative study of small organic compounds, called metabolites, in a biological specimen, is gaining more and more importance in nephrology research. Conducting a metabolomics study in human kidney disease cohorts, however, requires thorough knowledge about the key workflow steps: study planning, sample collection, metabolomics data acquisition and preprocessing, statistical/bioinformatics data analysis, and results interpretation within a biomedical context. This review provides a guide for future metabolomics studies in human kidney disease cohorts. We will offer an overview of important a priori considerations for metabolomics cohort studies, available analytical as well as statistical/bioinformatics data analysis techniques, and subsequent interpretation of metabolic findings. We will further point out potential research questions for metabolomics studies in the context of kidney diseases and summarize the main results and data availability of important studies already conducted in this field.

## 1. Introduction

Chronic kidney disease (CKD) has become one of the major global health burdens in the 21st century [1], with a typically chronic progressive disease course. Its extremely heterogeneous disease pattern and comorbidity load complicates the understanding of the underlying pathomechanisms and optimal patient treatment. Cohort studies form a suitable study design to investigate the associations between multiple exposures on the one hand and multiple outcomes on the other hand. They are particularly appropriate to study rare exposures or exposures for which randomization is not possible due to practical or ethical reasons. Even though randomized controlled trials (RCTs) are the gold standard for a research question of the effect of an exposure on an outcome, the majority of interventions investigated by RCTs in nephrology have so far been unable to demonstrate treatment benefits or have even caused harm [2]. This may well be due to the aforementioned heterogeneity of CKD populations. Large observational studies are therefore needed to appropriately characterize CKD population cohorts and identify interventionally treatable subgroups.

Important findings on kidney disease pathophysiology have already been accomplished by omics science, i.e., genomics [3,4], epigenomics [5], transcriptomics [6], and proteomics [7]. One of the latest additions to the omics research field is metabolomics, the quantitative study of small organic compounds, called metabolites, present in a biological specimen [8]. Metabolites are the intermediate and/or final products of molecular interactions between different proteins, signaling cascades, and cellular environments, thus constituting the end of the omics cascade. Additionally, they can arise from exogeneous sources, including food and drug intake, cosmetics, gut microbe–host co-metabolism, and others. The observation, analysis, and interpretation of the metabolites’ entirety, i.e., the metabolome, can therefore provide us with a metabolic “snapshot” or “fingerprint” of the current state of an organism. The ability of metabolomics studies to provide deeper insights into fundamental disease pathomechanisms has already been demonstrated for numerous other chronic diseases, including diabetes [9,10], cardiovascular diseases [11], and cancer [12]. Metabolomics is increasingly recognized as a valuable tool in the field of nephrology [13]. The first important metabolomics studies investigated metabolites as uremic toxins [14]. The research field then shifted towards the identification of metabolites associated with the patient’s glomerular filtration rate (GFR) [15,16], to metabolic fingerprints of adverse patient events [17], and has now extended towards the understanding of the underlying mechanisms in CKD progression. Especially the latter two goals make prospective CKD cohorts with measurements of the important patient parameters, e.g., GFR, and metabolites available at multiple time points a prerequisite. A multitude of different study questions can be tackled by applying metabolomics and for each study question a fitting study design is required. Conducting extensive metabolomics studies in large-scale cohorts of CKD patients might therefore enable the elucidation of important, possibly causal molecular disease traits and, consequently, improve CKD patient treatment.

**Figure 1:**
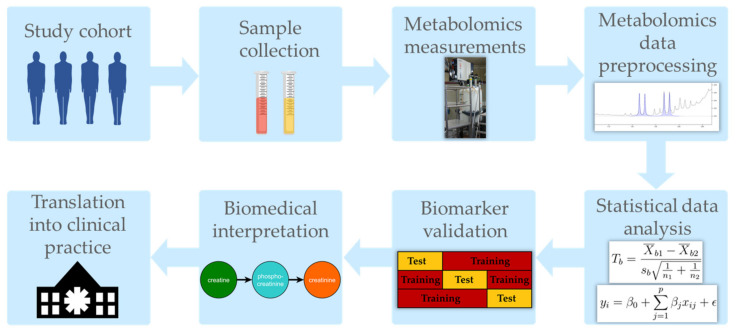
Schematic workflow of metabolomics studies in kidney disease cohorts.

This review provides a guide for future metabolomics studies in kidney disease cohorts using observational study designs. Conducting a thorough investigation of the metabolic changes related to impaired kidney function requires sophisticated study planning, metabolomics data acquisition and statistical/bioinformatics data analysis, as well as interpretation of the findings (Figure 1).

Important a priori considerations for metabolomics cohort studies, the available analytical as well as statistical/bioinformatics data analysis techniques, and subsequent interpretation of metabolic findings will be given. We will further point out potential research questions for metabolomics CKD studies and summarize the main results of important metabolomics studies already conducted in this field. A comprehensive summary of the metabolic markers of CKD discussed throughout the text is given in Table 1.

## 2. How to Get Started: A Priori Considerations for Metabolomics Cohort Studies

### 2.1. Possible Study Questions for Cohort Studies

**Figure 2:**
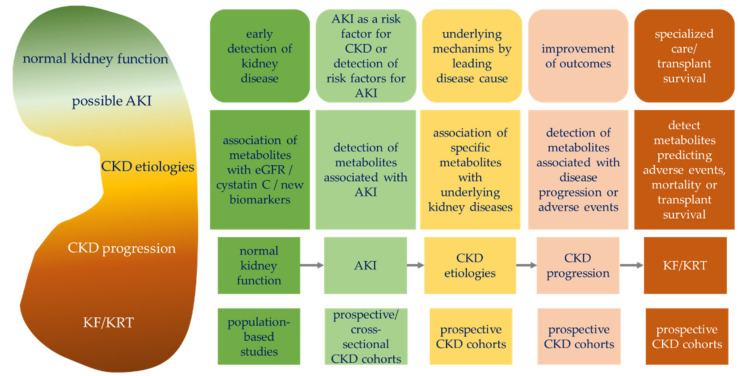
Possible research questions, kidney related prerequisites, and study cohort types used for metabolomics studies. Abbr.: AKI, acute kidney injury; CKD, chronic kidney disease; eGFR, estimated glomerular filtration rate; KF, kidney failure; KRT, kidney replacement therapy.

Before getting started with a metabolomics study in the field of nephrology, the researcher has to determine a study question of interest. Several exemplary study questions can be found in Figure 2, where some of the asked questions have already been investigated in the past.

Since an eGFR decline only occurs after a patient has already lost a considerable amount of kidney function, an early detection of kidney disease is a relevant study question. However, it can only be tackled within a population-based study including participants prior to a manifested diagnosis of CKD. For example, Sekula et al. were able to apply a non-targeted metabolomics approach within a population-based study (KORA F4) by associating metabolites with eGFR and were able to replicate 54 significantly associated metabolites in an independent cohort (Twins UK) [35]. CKD patients are more prone to acute kidney injury (AKI) events, but to elucidate the mechanisms of AKI, these patients have to be monitored more tightly than what a usual study design for cohort studies will request. So, nesting a sub-cohort of AKI patients within a prospective CKD cohort should be the way to go. Examples of small pilot studies with a limited number of participants to detect metabolites associated with and/or diagnostic of AKI can be found in [18,19,20]. Large prospective CKD cohorts offer the possibility to not only elucidate the general mechanisms of CKD, but to also delve deep into the causal pathways of differing CKD etiologies. Some examples of single, specific kidney diseases can be found in the literature, but within CKD cohorts larger patient populations with more power to detect the associations between the metabolites and kidney diseases can be collected. Recent advances in the metabolomics field have, for example, identified dysregulated energy metabolism between early- and late-stage diabetic kidney disease (DKD), a well-defined etiological CKD group, and elucidated the interaction between metabolic stress, mitochondrial homeostasis, and organelle crosstalk in the kidney as being important for dynamics during DKD progression [38]. Other examples can be found for membranous nephropathy, focal segmental glomerulosclerosis or IgA nephropathy [39], as well as autosomal dominant polycystic kidney disease (ADPKD) [21]. One of the big study questions is the identification of metabolites associated with or predicting CKD progression. Here, prospective CKD cohorts with measurements of kidney function markers and metabolites at multiple time-points during the study’s follow-up phase pose an invaluable treasure of information that only needs to be launched. Studies that have detected metabolites associated with CKD progression or with a higher risk of adverse patient events are, e.g., studies by Rhee et al. [14], Kalim et al. [24], or Zacharias et al. [17]. Rhee et al. identified metabolite alterations associated with subsequent disease progression, Kalim et al. showed that acylcarnitine may be associated with a higher uremic cardiovascular risk, and Zacharias et al. identified a multivariate metabolite signature for end-stage kidney disease (ESKD) risk prediction. In prospective ESKD cohorts, interesting research questions will then move towards the detection of metabolites to predict adverse events for hemodialysis patients or towards transplant survival/early detection of allograft rejection in kidney transplant patients. Some smaller studies with few participants investigating allograft rejection have been carried out in the past, e.g., studies by Blydt-Hansen et al. [22] and Suhre et al. [33]. These studies concluded the utility of metabolomics for non-invasive diagnosis of allograft rejection.

Besides prospective CKD cohort studies, other common study types can be applied in metabolomics studies of CKD, which are briefly discussed in the following paragraphs.

### 2.2. Common Study Designs in Human Cohorts

#### 2.2.1. Case Reports and Case Series

Historically, case reports, focusing on a single subject, or case series, reporting on a small group of phenotypically similar subjects, are a first step in identifying a new disease or adverse health effect from an exposure [40]. The possible association between the observed outcome and a specific exposure is described based on a small group of subjects. Such studies may be the first in identifying the value of a new scientific approach to clarify the pathophysiological background of a known disease [41]. Studies evaluating metabolomics in CKD started out small. For example, Shah et al. investigated only 30 participants with CKD and were able to show differences in the metabolic profiles for various CKD stages, reflecting alterations in arginine metabolism, elevated coagulation/inflammation, impaired carboxylate anion transport, and decreased adrenal steroid hormone production [25]. This study was a proof-of-concept study, setting the stage for large-scale prospective cohort studies in metabolomics of CKD. Another important small-scale proof-of-concept study of metabolic biomarker detection was conducted by Gronwald et al. [21]. Based on urinary nuclear magnetic resonance (NMR) metabolic fingerprints, the authors were able to discriminate ADPKD patients with moderately advanced disease from ADPKD patients with ESKD, patients with CKD of other etiologies, and healthy controls.

#### 2.2.2. Cross-Sectional Study

In a cross-sectional study, outcome and exposures are analyzed at the same time. In comparison to case–control (participants selected based on the outcome status) or cohort studies (participants selected based on the exposure status), the participants in a cross-sectional study are only selected based on the inclusion and exclusion criteria. This type of study design can be easily implemented, is rather cheap, and can be started at enrollment or any later time point during the course of a cohort study. Since the inference of causal relationships between exposure and outcome by a one-time measurement is not possible, these studies are traditionally used to investigate disease prevalence or the influence of environmental factors such as drugs, toxins, or diet, as, e.g., demonstrated in [42].

Cross-sectional studies can further be employed to identify subgroups or stages in complex diseases. Luo et al., for instance, identified 58 serum metabolites associated with proteinuria in a cross-sectional study design, some of which were also associated with CKD progression [37]. Within a proof-of-concept study setting, multivariate metabolite signatures of measured GFR were used to improve GFR estimation [26]. Goek et al. found the serum concentrations of spermidine to be associated with kidney function change in the general population, and serum metabolites were able to predict incident CKD [34]. Another study by Barrios et al. investigated metabolic signatures of diabetic nephropathy combining four European cohorts [43].

#### 2.2.3. Case–Control Study

Within case–control studies, the odds of an exposure within a predefined group with a characteristic trait of interest are compared to the odds of an exposure in a control group. When appropriately designed, case–control studies (1) can provide the same information as a cohort study; (2) are more rapid and efficient, because, unlike in cohort studies, only a minority of the population is included in the study; and (3) data on exposure are being collected in retrospect. Disadvantages include: (1) ‘general types of bias’; (2) specific sources of bias; and (3) selection of cases and controls can prove to be complex [40]. A special form of case–control study is the nested design of a case–control study, where cases and controls are drawn from within a prospective study. All cases who developed the outcome of interest during the follow-up are selected and compared with a random sample of the cohort [41]. A nested case–control study of metabolomics in a CKD population was, for instance, carried out within the Chronic Renal Insufficiency Cohort (CRIC) Study by Rhee et al. in 2016 [14]. The authors compared a subset of the CRIC Study population with rapid progression of kidney disease according to eGFR slopes to a subset with slow progression. For each case a control was selected that was categorized within the same eGFR and proteinuria category at study entry. Ten metabolite alterations were nominally associated with subsequent CKD progression and, cross-sectionally, six of the metabolites that were higher in the cases than controls were significantly associated with eGFR at baseline. The authors concluded that their results warrant further interest in arginine, methionine, and threonine as potential markers of kidney function and progression of kidney disease.

#### 2.2.4. Prospective Cohort Study

As already outlined above, prospective cohort studies collect consecutive information on outcomes and exposures from the same participants within a specific time period. They allow, e.g., time-to-event analyses, time-course evaluations, and risk score development based on metabolite measures. To date, only few prospective CKD studies acquired metabolomics data, amongst them the German Chronic Kidney Disease (GCKD) study [44]. Within this study, Zacharias et al. developed a novel risk score based on NMR-derived plasma metabolic features, including creatinine, high-density lipoprotein, valine, acetyl groups of glycoproteins, and Ca^2+^-EDTA, to predict the risk of ESKD within four years after the metabolomics measurements [17]. In another metabolomics study conducted in the GCKD cohort, Sekula et al. discovered a significant association between higher urinary 6-bromotryptophan levels and lower risk of kidney failure, both unadjusted and adjusted for kidney failure risk factors other than eGFR [28]. Similar results were obtained for investigations of serum 6-bromotryptophan levels. Steinbrenner et al. discovered 55 urinary metabolites that predict adverse kidney outcomes and/or mortality, including C-glycosyltryptophan, within a metabolome-wide association study [29]. Within the same cohort, Schlosser et al. identified the underlying molecular mechanisms related to the absorption, distribution, metabolism, and excretion (ADME) of metabolites in the kidney [31]. Comprehensive summaries of recent ongoing prospective CKD cohort studies within the International Network of Chronic Kidney Disease cohort studies (iNET-CKD) can be found in [45]. Another example from the CRIC Study by Kwan et al. detected a negative association of 3-hydroxyisobutyrate and 3-methylcrotonylglycine with eGFR slopes whereas citric acid and aconitic acid were positively associated.

#### 2.2.5. Randomized Controlled Trial

Since RCTs, when carried out appropriately, are still the gold-standard for studying the effects of an intervention or any other type of therapy on an outcome, RCTs in a CKD metabolomics context might likewise be of interest. The first steps into this direction have been taken in animal studies as well as for other disease entities. Hypertension in CKD patients, one of the leading underlying CKD causes, is mostly treated via several available drugs, but another treatment strategy could be to metabolically rewire the hypertensive kidney. Rinschen et al. were able to show promising results in animal models, leading to possible future dietary intervention studies [46]. These kinds of studies would constitute a metabolic challenge that can be supervised by measuring metabolites before and after the intervention of, e.g., a lipid-consuming, ketogenic diet in comparison to controls. Similar studies have been carried out with dietary interventions in patients diagnosed with rheumatoid diseases [47], as well as diabetes [48]. In the latter, postprandial metabolic alterations in healthy men with a high genetic risk of diabetes were evaluated after two meals with a varying macronutrient content, finding that modifications in intermediate lipid metabolism were induced by a high caloric meal.

### 2.3. Important Considerations for Sample Collection in Metabolomics Studies

In human studies involving non-deceased study participants, common specimen types include plasma, serum, urine, whole blood, saliva, cerebrospinal fluid, feces, and tissue. Plasma, serum, and urine, in particular, constitute the most suitable biofluids analyzed by metabolomics in large-scale cohorts due to easy sample collection, handling, and preparation, as well as being in high abundance, and, most importantly, involved in key renal regulatory mechanisms. The analysis of kidney tissue is likewise important, but requires more complex, invasive sample collection and extensive sample preparation.

Sample collection should, ideally, be carried out in a standardized fashion according to well-defined standard operating procedures (SOPs) across the whole study period to minimize unwanted technical and biological sample variation. The metabolite content of a blood and urine specimen is significantly influenced by an individual’s fasting status, fluid intake, circadian rhythm, age, sex, body fat composition, comorbidities, and specific lifestyles, including smoking and alcohol intake, as well as a plethora of different medications [49,50,51], but also by numerous genetic factors [52]. The collection of such biofluid specimens should thus be carried out within a homogeneous time window across the whole study cohort, ideally after a well-defined fasting state period. To appropriately account for non-influenceable confounders, e.g., sex or comorbidities, matching or randomization strategies should be applied, and/or confounder adjustment and stratification during the statistical analysis. Here, accurate documentation of all important phenotypical, but also study protocol information, such as sample collection time, is warranted. To avoid bacterial growth in freshly collected urine samples, appropriate preservation steps, i.e., either filtration, centrifugation, or addition of bacteriostatics, should be carried out [53]. In the case of plasma samples, the use of only one specific type of anticoagulant, e.g., ethylenediaminetetraacetic acid (EDTA), heparin, or citrate, across the whole sample cohort is strongly recommended, since substance traces can appear in metabolic fingerprints and might complicate further analysis steps [19,54]. Ideally, appropriate sample volumes should be immediately aliquoted for subsequent metabolomics measurements to avoid unnecessary freeze–thaw cycles. To ensure metabolite stability, samples should be frozen at −80 °C immediately after collection until further processing. Especially in the case of large-scale cohort studies comprising hundreds to thousands of individual specimens, automatic sample handling and documentation is strongly recommended. A comprehensive review, including the SOPs for optimal pre-analytical handling of, e.g., urine, plasma, serum, and tissue specimens for subsequent metabolomics measurements, is provided in [53].

## 3. Metabolomics Data Acquisition

### 3.1. Common Analytical Platforms in Metabolomics Studies

Two main analytical platforms are commonly used for metabolomics studies: nuclear magnetic resonance (NMR) spectroscopy and hyphenated mass spectrometry (MS). The principle of NMR spectroscopy is based on the separation of different analyte signals by their resonance frequencies within a magnetic field. It is particularly well suited for large-scale metabolomics studies, since instrumentation and data acquisition is highly stable across time and even across different lab facilities [55]. Only few, rather cheap sample preparation steps are mandatory and no metabolite derivatization is needed. Due to its non-destructive nature, NMR experiments allow the re-use of sample material after measurement and instrument cleaning is not required. NMR spectroscopic data allows, theoretically, the absolute quantification of all detectable metabolites with the use of only one internal standard. However, NMR spectroscopy suffers, in comparison to hyphenated mass spectrometry, from low sensitivity, resulting in lower metabolite coverage. Due to typically limited time resources, only one-dimensional (1D) NMR experiments are carried out for large-scale cohort studies. 1D NMR spectra, especially of urine and plasma/serum specimens, exhibit a high number of spectrally overlapping metabolite signals, which might complicate subsequent metabolite identification and accurate quantification. Two-dimensional (2D) NMR experiments are able to resolve these strongly overlapping signals into a second dimension and can provide further structural information about the detected metabolites, enhancing metabolite identification. Significantly longer acquisition times for 2D NMR experiments, however, preclude their wide application for large-scale cohort studies, although recent progress in the development of, e.g., non-uniform sampling techniques for 2D NMR [56] might overcome this obstacle soon. Although NMR experimental costs are, in general, low, the initial set-up of a well-operating NMR spectroscopy platform suitable for high-throughput metabolomics measurements is expensive and specific site requirements have to be fulfilled. Commercial NMR metabolomics platforms have been established in recent years and have proven their reliability in numerous studies [57]. The latest instrumental and analytical developments include the miniaturization of NMR spectrometers to a “benchtop” size [58,59,60] and the introduction of Bruker IVDr methods [61,62].

In contrast, hyphenated mass spectrometry, such as liquid chromatography (LC) or gas chromatography (GC)–MS offer much higher sensitivity and selectivity. MS identifies metabolites according to their mass-to-charge-ratios. It is typically coupled to an LC or GC, which separate analytes according to different physical and chemical properties, e.g., molecular size, charge, polarity, and affinity toward other molecules [63]. In contrast to NMR, which requires about 100–400 µL volume per biofluid specimen [62,64], MS experiments are typically carried out with much lower sample volumes of about 10 µL. MS sample preparation usually includes a derivatization step and the addition of individual internal standards for each absolutely quantified metabolite. These sample preparation steps, but also specific sample introduction systems and ionization techniques can prevent the detection of certain metabolite classes [65]. MS techniques are per se destructive and samples cannot be recovered after measurement. However, due to the low sample volume required, this hardly ever constitutes a serious limitation for MS in human cohort studies. The initial installation of a hyphenated MS system is, in comparison to an NMR spectrometer platform, cheaper, and less elaborate site requirements have to be fulfilled. On the other hand, hyphenated MS systems are, in general, less robust, and therefore data are less reproducible than when acquired on NMR systems; MS systems also require regular instrument cleaning. It has to be noted that the metabolome coverage of NMR spectroscopy and hyphenated MS, although displaying very good overlap between the different techniques, still exhibits distinct differences [66], and these analytical platforms should be rather considered as complementary than competing. Instrumental improvements in hyphenated mass spectrometry include the introduction of comprehensive two-dimensional (2D) gas chromatography (GC x GC), displaying superior separation capacity for complex biological mixtures, high sensitivity, peak resolution, and reproducibility [67]. Likewise, comprehensive 2D LC x LC substantially reduces peak overlap [68]. Imaging mass spectrometry (IMS) enables the in vivo or in vitro detection and 2D or 3D imaging of metabolites in tissues or cells and thus provides additional spatial information about metabolite distributions in these specimens [69].

Irrespective of the employed analytical platform, metabolomics analyses can be conducted in two different approaches: targeted or untargeted metabolomics. Targeted metabolomics constitutes the accurate detection and often absolute quantification of a preselected set of known metabolites. Commercially optimized kits for high-throughput quantitative analysis are readily available and several commercial contract research organizations offer targeted and/or untargeted metabolomics measurement services [70]. Such targeted MS protocols allow high-throughput measurements with excellent reproducibility. Non-targeted metabolomics, in contrast, aims at maximization of metabolome coverage without any a priori metabolite selection, i.e., hypothesis free. Analyte signals of interest, typically revealed by statistical data analysis, are then identified post hoc. For MS techniques, non-targeted metabolomics is only able to provide semi-quantitative metabolite measures since individual internal standards are naturally missing. NMR spectroscopy, however, still allows a posteriori absolute metabolite quantification after accurate identification of previously unknown metabolites measured in an untargeted approach. The choice of metabolomics approach for a nephrological study should be based on the particular research question: if the study aims at elucidating the role of one or several distinct metabolites in a phenotype, which are known based on previous research, a targeted, hypothesis-driven approach is recommended. If the study aims at uncovering yet unknown metabolic key players involved in a specific phenotype, an untargeted, hypothesis-generating approach should be employed.

### 3.2. Sample Preparation, Measurements, and Preprocessing in Metabolomics Studies

Numerous comprehensive protocols for both NMR and hyphenated MS, including elaborate sample preparation, measurement, and data preprocessing workflows, are available [8,63,64,71,72,73,74]. A selective summary of the key metabolomics data preprocessing steps, including the available software tools, is provided in Table 2. In brief, sample preparation for NMR-based metabolomics studies includes the addition of buffer solution, D_2_O, and a spectral reference substance, such as 3-trimethylsilyl-2,2,3,3-tetradeuteropropionate (TSP), to the respective urine, plasma, serum, or tissue extract specimens [64]. Please note that the protein present in the specimen, as, for instance, in plasma or urine of patients suffering from proteinuria, gives rise to broad, unspecific NMR signals, which might obscure smaller metabolite signals, and severely binds to the reference substance TSP [64]. In this case, TSP can no longer be used as a reference for absolute quantification, and other reference substances, e.g., formic acid, have to be employed [75]. Alternatively, proteins can be removed prior to metabolomics data acquisition by, e.g., ultrafiltration or chemical protein precipitation [76], or a specific NMR pulse sequence, the Carr–Purcell–Meiboom–Gill (CPMG) sequence can be employed to suppress broad protein signals [75]. Likewise, suitable water suppression techniques are typically employed during NMR data acquisition for urine, plasma, and serum specimens to reduce the dominance of the strong water signals in the spectra [70]. Preprocessing of raw NMR data includes Fourier transformation of the NMR signal with the application of an exponential filter function, as well as phase and baseline correction [64]. To facilitate statistical evaluation of NMR spectra, the corresponding NMR signals need to be extracted beforehand. Various NMR signal extraction methods have been proposed, but a simple binning of the complete spectrum into equidistant sections of, e.g., 0.01 ppm width, is probably still the most popular technique [8]. Any metabolomics dataset is affected by unwanted technical and/or biological variances and biases, such as varying dilution of urine specimens [8]. These variances can be reduced by appropriate data normalization techniques, but subsequent statistical analysis results are inherently dependent on the specific, a priori chosen method [8,77]. To overcome this issue, Zacharias et al. proposed the application of (logistic) zero-sum regression [78,79] for the generation of normalization-invariant multivariate metabolic biomarker signatures, which proved to yield highly robust and predictive metabolic biomarker signatures of AKI after cardiac surgery [77]. Besides data normalization, both NMR and MS metabolomics data are typically transformed to approximately follow a multivariate normal distribution and to exhibit constant variance, e.g., by application of a log transformation [8]. In the case of untargeted NMR metabolomics studies, statistical analysis steps are typically carried out with yet unidentified NMR spectral features, and subsequent metabolite identification only focuses on statistically relevant NMR peaks. This identification is achieved by manual comparison of the complex NMR spectrum of a biofluid specimen to the NMR reference spectra of pure compounds, available from either commercial or public data bases, e.g., the Human Metabolome Data Base (HMDB) [64,80]. Here, additional 2D NMR measurements can offer extremely valuable structural information to support this identification step. Following successful identification, these metabolites can then be absolutely quantified [64].

For LC-MS measurements, removal of protein during the sample preparation step, e.g., by methanol extraction, is mandatory to avoid signal suppression of the low-abundance analytes and protein precipitation under reversed-phase LC conditions [70]. The analysis of urine by LC-MS techniques is challenged by the high salt content, varying dilution, and the complex composition of the samples [70]. Various analytical pretreatment and data normalization strategies have been proposed to overcome these issues [81]. A systematic comparison of different protocols by Vogl et al. revealed that dilution of urine specimens to a fixed creatinine concentration yielded the least number of missing values and allowed reliable classification of urine specimens from healthy controls and CKD patients [81]. The urinary creatinine concentration is, however, significantly influenced by sex, age, muscle mass, diet, pregnancy, and renal pathology [82,83,84,85]. Alternative normalization approaches have thus been proposed, including a normalization to the urine volume, osmolality, and “total useful MS signal” [86,87]. Since, however, the choice of MS data normalization strategies, as described above in an analogous manner for NMR data normalization strategies, substantially influences subsequent statistical data analysis results [86], it is recommended to either employ a combination of different normalization strategies [86,87] or normalization-invariant data analysis methods, such as zero-sum regression [77,78]. The application of GC-MS for metabolomics analyses requires the volatilization of the analyzed compounds, which have to be thermally stable. Subsequent data preprocessing steps again include feature extraction and (automatic) identification, based on commercial or freely available databases, as well as absolute quantification of metabolites. One should keep in mind that the latter is only possible if the corresponding internal standard had been included in the measurement step [70]. Both untargeted and targeted MS datasets include certain amounts of missing data points due to failed peak detection, leading to incomplete data matrices. Since many statistical data analysis methods, however, require complete data matrices, these missing data points are typically imputed prior to statistical analysis. A combination of NMR and hyphenated MS experiments can significantly enhance the metabolite identification in untargeted metabolomics studies.

## 4. Statistics and Bioinformatics Data Analysis

Probably the main goal of metabolomics analyses in biomedical research is the detection of powerful metabolic biomarkers for disease diagnosis or prognosis, response to therapeutic interventions, or, in general, response to external stimuli, e.g., nutrition or exercise. The NIH defines the term “biomarker” as “a characteristic that is objectively measured and evaluated as an indicator of normal biological processes, pathogenic processes, or pharmacologic responses to a therapeutic intervention” [141]. The search for novel metabolic biomarkers in the context of nephrology, as illustrated in Figure 2, is a highly emerging research area. From a statistical point of view, several different approaches can be distinguished (Table 3):

• **Hypothesis testing:** Univariate statistical differentiation between two or more predefined groups.
• **Multivariate biomarker signature detection:** Generation of multivariate regression scores to predict an outcome of an unknown test sample.
• **Subgroup identification:** Exploratory approach to identify biomedically different patient/sample subgroups.
• **Metabolome-wide association study:** Systematic analysis of the entire measured metabolome based on regression, including appropriate confounder adjustment to identify significant associations between metabolites and an outcome. A correction for multiple testing is essential for these comparisons.
• **Statistical network analysis:** Systematic analysis of interactions between different metabolites and/or patient parameters, other omics variables, etc., which are represented as a network. Allows a holistic view on the metabolome and its interaction with specific phenotypes, and can reveal molecular mechanisms or regulating processes.
• **Meta-analysis:** Combination of statistical results across multiple studies to increase statistical power and to gain more robust results.
• **Time-to-event analysis:** Time-to-event data contain information about if and when an event occurred, but typically also censored data. Survival analysis appropriately associates time-to-event data with, e.g., metabolite levels.
• **Time-course analysis:** Analysis of metabolite concentration changes across time and typically in response to external stimuli.
• **Pathway (enrichment) analysis:** Post-hoc mapping of differential metabolites to metabolic pathways, employing pathway databases, e.g., KEGG [142] or Gene Ontology [143], and subsequent testing if significantly differentiating metabolites are significantly enriched in a specific pathway.

The statistical analysis of high-dimensional metabolite data often includes multiple comparisons, which easily can result in a high number of false positives. To reduce this error and avoid misleading conclusions, the *p*-values have to be corrected for multiple testing by, e.g., adjustment of the false-discovery rate (FDR) as proposed by Benjamini and Hochberg [144]. Since the statistical analysis of metabolomics data requires a broad range of different methods, alongside the popular statistical analysis software *R* [145], several stand-alone software solutions exist, providing a collection of web-based tools with graphical user interfaces, e.g., MetaboAnalyst [113] and 3Omics [146], as reviewed by Cambiaghi et al. [147].

## 5. Validation, Interpretation, and Beyond

Any statistical results of metabolomics studies have to be carefully validated. Ideally, novel statistically significant metabolic biomarkers should prove to be still significantly associated with the respective outcome in independent cohorts. Since metabolomics data in large-scale human CKD cohorts are still scarce, possibilities to replicate, e.g., a significant association between a set of metabolites and time-to-kidney-failure, are rather limited. Likewise, the predictive performance of novel metabolic risk scores for the diagnosis or prognosis of specific renal outcomes has to be validated on independent test sets to proof any clinical utility. Luckily, several strategies for unbiased performance assessment of novel classification or prediction scores within the same patient cohort are well established, e.g., cross-validation, where the complete data set is iteratively split into training and test data sets and, within each cross-validation run, the new predictive model is solely trained on the training set and solely tested on the test set, respectively (compare to Figure 1) [190].

Next to statistical replication, further exploration of metabolic findings, discovered in human cohorts, can be carried out in suitable animal models or cell lines, and vice versa. Animal and cell line studies offer the huge advantage of a very controlled experimental environment and suitability for extensive intervention studies, and they are able to further elucidate the underlying pathophysiologic mechanisms [191]. Chen et al., for example, identified 5-methoxytryptophan (5-MTP) as a potential marker of CKD in a human cohort, and subsequently examined the anti-inflammatory and anti-fibrotic effects of 5-MTP and the biological roles of its regulatory enzyme tryptophan hydroxylase-1 in cell and animal models [27]. A next step typically carried out in metabolomics studies is the interpretation of metabolic biomarkers in the context of their metabolic pathway environment, also known as pathway mapping, as well as in the context of already published research. Numerous open-source software for pathway mapping exist, including MetaboAnalyst [113], as extensively reviewed in [192]. The explosively growing amount of metabolomics data from many small studies and different analytical platforms, however, challenges the unified interpretation of metabolic findings across different studies. Abbiss et al. provide an extensive list of metabolites that have been reported as important for two or more kidney diseases [193].

The interplay of the microbiome and the metabolome in terms of the gut–kidney axis and its contribution to kidney diseases is reviewed in [194] and might help to highlight common biochemical processes in kidney diseases, such as the purine and tryptophan metabolism. The Human Metabolome Database (HMDB; https://hmdb.ca/ (accessed on 10 June 2021)) offers a rich source of information on metabolites, their chemical properties, normal and abnormal abundances, biochemical/enzymatic/pathway data, as well as important literature references [80]. Kidney-specific web resources for different omics data, including Nephroseq (https://www.nephroseq.org/ (accessed on 10 June 2021)), the Kidney and Urinary Pathway Knowledge Base (KUPKB; www.kupkb.org (accessed on 10 June 2021)) [195], and the Chronic Kidney Disease database (CKDdb; www.padb.org/ckdbd (accessed on 10 June 2021)) [196], which allow the unification of all available information from different sample origins and omics levels, are reviewed by [197].

While metabolomics studies represent a fascinating research field with huge potential that still needs to be launched on its own, an integration of multiple omics datasets will further help to elucidate CKD pathomechanisms. Multi-omics studies will make use of genome-wide association study (GWAS) data, whole exome or whole genome sequencing from DNA, messenger RNA (mRNA) as the product of gene transcription, as well as proteomics and metabolomics from the same patient or even the same sample. Genomic analyses can identify the risk factors/disease causing variants and can thereby enlighten regulatory networks. Together with proteomics and metabolomics measurements, one will be able to delve deeper into a functional/molecular basis of disease pathology [198]. Network analyses exploring the interconnectivity of genetic and molecular entities in CKD will provide additional information on the critical drivers of kidney diseases. Moreover, these networks will expand our understanding of how CKD affects different body systems and how stimuli, such as diet, medication, and the microbiome, participate in this complex interplay [199]. Together, this will bring the field of omics research closer to possible clinical applications in order to improve patient treatment. Metabolomics especially has great potential for large-scale utilization in clinical practice; however, its current application in clinical routines is still limited. Current obstacles, which have to be resolved, include the development of small-scale measurement devices; extensive validation in external cohorts; introduction of SOPs for sample collection, storage, preparation, measurement, and preprocessing; data analysis and interpretation; and unambiguous metabolite identification as a key prerequisite for the development of targeted measurement kits [200,201].

## 6. Conclusions

The field of metabolomics already has been of unmeasurable value for nephrology research. Still, many questions remain and need to be addressed in the future. A first issue will be to understand the differing metabolite patterns across the diverse spectrum of kidney diseases, such as metabolic syndrome/diabetes mellitus, glomerular diseases, and many others; but, within similar phenotypic CKD etiologies, metabolomics also will help to unravel the mechanisms that differentiate, e.g., slow from fast CKD progressors. Translation of metabolomics research into routine CKD patient care will pave the way for novel metabolic biomarkers to evaluate and monitor the efficacy or safety of patient treatments. Thus, metabolomics studies will support clinical decision making. Eventually, metabolomics will become an integrated part of CKD diagnostics and will be able to inform the treating physicians on the rate of CKD progression, adverse risk evaluation, and other CKD-related comorbidities, such as the stage of metabolic syndrome vs. diabetes mellitus or others. Thereby, metabolomics will be a pioneering field for individualized patient treatment.

## Figures and Tables

**Table 1:** Important metabolite biomarkers of chronic kidney disease and its sequelae. Abbr.: AASK, African American Study of Kidney Disease and Hypertension study; ADPKD, autosomal dominant polycystic kidney disease; AKI, acute kidney injury; ARIC, Atherosclerosis Risk in Communities; ArMORR, Accelerated Mortality on Renal Replacement study; CKD, chronic kidney disease; CRIC, Chronic Renal Insufficiency Cohort; CTOT-04, Clinical Trials in Organ Transplantation 04 Study; CVD, cardiovascular disease; DKD, diabetic kidney disease; eGFR, estimated glomerular filtration rate; FSGS, focal segmental glomerulosclerosis; GCKD, German Chronic Kidney Disease; HDL, high-density lipoprotein; IDL, intermediate density lipoprotein; IgAN, IgA nephropathy; KORA, Cooperative Health Research in the Region of Augsburg; KRT, kidney replacement therapy; LDL, low-density lipoprotein; MDRD, Modification of Diet in Renal Disease; MESA, Multi-Ethnic Study of Atherosclerosis; mGFR, measured glomerular filtration rate; MGN, membranous glomerulonephritis; TCMR, T cell-mediated rejection; UACR, urinary albumin-to-creatinine ratio.

Study Design/Type	Study Question	Study Population and Investigated Biofluids	References	Detected Metabolites/Metabolic Biomarkers/Pathways
case–control study	AKI prediction	patients undergoing cardiac surgery, urine specimens collected before and after surgery	[18]	carnitine (elevated in AKI-free patients), tranexamic acid (elevated in AKI patients) and others
case–control study	AKI prediction	patients undergoing cardiac surgery, plasma specimens collected 24h after surgery	[19]	glucuronide conjugate of propofol, Mg^2+^, lactate and others
case–control study	indicators of AKI	hospitalized, newly diagnosed AKI patients, serum specimens	[20]	increases in acylcarnitines and amino acids and reduction of arginine and lysophosphatidyl cholines in AKI patients
case–control study	distinct metabolic profile of ADPKD	54 patients with ADPKD, several control groups, urine specimens	[21]	on average 51 out of 701 NMR features could reliably discriminate ADPKD patients from other kidney disease patients and healthy controls
case–control study	non-invasive diagnosis of TCMR in pediatric kidney transplant recipients	pediatric kidney replacement recipients, urine specimens	[22]	proline, kynurenine, phosphatidylcholines, diacylglycerols elevated in TCMR patients
case–control study	identify metabolic pathways altered in CKD stage 3–4 non-diabetics	CKD patients from the Paricalcitol study; healthy controls: employees of study centers, urine and plasma specimens	[23]	27 urine and 33 plasma metabolites differed between CKD vs. controls; pathway analysis: citric acid cycle significantly affected: reduction of urinary excretion of citrate, cis-aconitate, isocitrate, 2-oxoglutarate, succinate; expression of genes regulating these metabolites were reduced
2 independent nested case–control studies (=analysis vs. replication cohort)	metabolites predicting CVD mortality in incident KRT patients	ArMORR study, plasma specimens	[24]	oleoylcarnitine, linoleoylcarnitine, palmitoylcarnitine, stearoylcarnitine, strongest association with CVD mortality: oleoylcarnitine
cross-sectional CKD study	plasma metabolite profile differences in CKD stages 2, 3, and 4	30 participants with differing CKD stages, plasma specimens	[25]	CKD stages 3 vs. 2: 62 differing metabolites (39 higher and 23 lower in CKD stage 3); CKD stages 4 vs. 2: 111 differing metabolites (66 higher and 45 lower in CKD stage 4); CKD stages 4 vs. 3: 11 differing metabolites (7 higher and 4 lower in CKD stage 4); major differences for higher CKD stages: altered arginine metabolism, elevated coagulation/inflammation, impaired carboxylate anion transport, decreased adrenal steroid hormone production
cross-sectional study (proof-of-concept study)	identfication of serum metabolites to provide a more accurate GFR estimate	AASK study, MESA study: participants with mGFR, serum specimens	[26]	(1) serum metabolites from untargeted quantification: AASK—283 and MESA—387 significantly associated metabolites with mGFR; (2) targeted metabolites: 15 metabolites used for GFR estimation
2 cross-sectional observational studies of the general population	association of serum metabolites and their ratios with eGFR	KORA F4 study, TwinsUK registry, serum specimens	[15]	association with eGFR: 22 metabolites and 516 metabolite ratios; acylcarnitines were associated inversely, ratio with the lowest *p*-value: serine to glutarylcarnitine
differing study design per cohort	metabolites correlating with clinical markers of kidney disease	4 cohorts: training cohort, validation cohort, prospective cohort, drug treatment cohort	[27]	5 metabolites, e.g., 5-metohydroxytryptophan, correlate with markers of kidney function
nested case–control study	CKD progression	CRIC study, serum specimens	[14]	10 nominally associated metabolites; 6 higher in cases (uric acid, glucuronate, 4-hydroxy-mandelate, 3-methyladipate/pimelate, cytosine, homo-gentisate) and 4 lower in cases (threonine, methionine, phenylalanine, arginine)
prospective CKD cohort	risk of progression to KRT	GCKD study, plasma specimens	[17]	24 NMR features—highest weights: creatinine, high-density lipoprotein, valine, acetyl groups of glycoproteins, Ca^2+^-EDTA
prospective CKD cohort	urinary 6-bromotryptophan and incident ESKD	GCKD study, urine specimens	[28]	higher 6-bromotryptophan levels were associated with lower risk of ESKD
prospective CKD cohort	urine metabolites associated with adverse kidney outcomes and mortality	GCKD study, urine specimens	[29]	55 metabolites significantly associated with kidney failure, kidney failure + AKI or death; significant enrichment for phosphatidylcholine pathway
prospective CKD cohort	adverse cardiac events in CKD stage 3 patients	GCKD study, plasma specimens	[30]	association of trimethylamine *N*-oxide (TMAO) with cardiac arrhythmia and myocardial infarction
prospective CKD cohort, prospective population-based cohort	genetic studies of urinary metabolites	GCKD study, UK Biobank, urine specimens	[31]	240 unique metabolite-locus associations highlighting novel candidate substrates for transport proteins; genes identified are enriched in absorption, distribution, metabolism, and excretion (ADME) relevant tissues, potentially novel candidates for biotransformation and detoxification reactions
prospective diabetic cohort study	multimetabolite models of disease process from type 1 diabetic patients w/o CKD	Finnish Diabetic Nephropathy Study Group, serum specimens	[32]	cross-sectionally: patients w/o DKD complications: low lipids, less inflammation, better glycemic control vs. patients with advanced CKD: high sphingomyelin, cystatin-C; shared features: low unsaturated fatty acids (UFA), phospholipids; prospectively: progressive albuminuria: high UFAs, phospholipids, IDL, LDL; accelerated DKD progression: high saturated fatty acids, low HDL
prospective observational transplant recipient study	prediction of allograft status via urine metabolites	kidney graft recipients of the CTOT-04 study, urine specimens	[33]	best discrimination between acute cellular rejection vs. no rejection: ratio of urinary 3-sialyllactose to xanthosine
prospective population-based study	metabolite associations with eGFR; incident CKD	ARIC study, serum specimens	[16]	eGFR associations: 34 metabolites detected—strongest positive = creatinine, strongest negative = 3-indoxyl sulfate; lower risk of incident CKD: 5-oxoproline, 1,5-anhydroglucitol
prospective population-based study	kidney function decline, incident CKD	KORA S4/F4 study, serum specimens	[34]	kidney function decline: spermidine, phosphatidylcholine diacyl C42:5-to-phosphatidyl acyl-alkyl C36:0 ratio; incident CKD: kynerunine-to-tryptophan ratio
prospective population-based study; prospective twin cohort	metabolite association with eGFR, incident CKD	KORA F4 study, replication in TwinsUK registry, serum specimens	[35]	54 metabolites replicated and significantly associated with eGFR; 6 with pair-wise correlation with established kidney function measures (C-mannosyltryptophan, pseudouridine, *N*-acetylalanine, erythronate, myo-inositol, *N*-acetylcarnosine); incident CKD: C-mannosyltryptophan, pseudouridine, O-sulfo-l-tyrosine
prospective small patient sample	metabolic changes after kidney allograft transplantation	19 allograft recipients, serum specimens	[36]	hippurate, mannitol, and alanine associate with changes in transplant allograft function over time; hippurate/histine are more sensitive to short-term changes in kidney activity than creatinine
two clinical trials	cross-sectional association of UACR with 637 known, non-drug, blood metabolites	AASK, MDRD study, serum specimens	[37]	58 metabolites associated with proteinuria; metabolites with lowest *p*-value: 4-hydroxychlorthalonil and 1,5-anhydroglucitol with all 6 metabolites of the phosphatidylethanolamine pathway being significant
review	DKD associated metabolites	multiple studies	[38]	early stages of DKD: association with tricarboxylic acid cycle, glucose metabolites; uremic toxins in DKD progression: phenyl sulfate and tryptophan derivatives
review	differential metabolites in MGN, FSGS, IgAN	multiple studies	[39]	amongst others—MGN: 13 urinary metabolites as most important (dopamine, fumarate, carnosine, nicotinamide d-ribonucleotide, pyridoxal, deoxyguanosine triphosphate, adenosine monophosphate, l-citrulline, nicotinamide, deoxyuridine, phenylalanine, tryptamine, succinate); FSGS: 10 prognostic urine metabolites (citrulline, proline, dimethylamine, acetoacetate, valine, alphaketoisovaleric acid, isobutyrate, histidine, d-palmitylcarnitine, *N*-methylnicotinamide); IgAN vs. controls: higher serum metabolite levels (phenylalanine, lactate, myo-Inositol, L6 lipids L5 lipids, L3 lipids) and lower serum metabolite levels (alpha-, beta-glucose, valine, phosphocholine, tyrosine, lysine, isoleucine, glycine, glycerolphosphocholine, glutamate, glutamine, alanine, acetate, 1-methylhistidine, 3-hydroxybutyrate)
perspectives, no study design	metabolomics in CKD research: metabolites and future risk of mortality	AASK study, serum specimens	[13]	number of associated metabolites reduced after adjustment for eGFR—metabolite classes detected: amino acid, carbohydrate, cofactors/vitamins, energy, lipid, nucleotide, peptide, xenobiotic, unkown

**Table 2:** Important preprocessing steps in targeted and/or untargeted metabolomics studies and selected commercial or freely available preprocessing software. Abbr.: JBA, pJRES binning algorithm; kNN, k-nearest neighbors; MCR-ALS, Multivariate Curve Resolution-Alternating Least Squares; MICE, multivariate imputation by chained equations; MS, mass spectrometry; NMR, nuclear magnetic resonance; RF, random forest; ROI, region of interest; SRV, statistical recoupling of variables.

Preprocessing Step	Goal		Available Methods		Commercially Available Software		Freely Available Software	
	NMR Spectroscopy	Hyphenated MS	NMR Spectroscopy	Hyphenated MS	NMR Spectroscopy	Hyphenated MS	NMR Spectroscopy	Hyphenated MS
spectral preprocessing	transform spectral data from time to frequency domain, correct baseline and phase distortions	reproducible identification and quantification of peak features across multiple MS spectra	Fourier transformation, zero filling, apodization, phase correction, baseline correction, spectral alignment, removal of unwanted regions	deisotoping, retention time alignment, baseline and noise filtering, recalibration	TopSpin (BrukerBioSpin GmbH, Rheinstetten, Germany), AMIX (BrukerBioSpin GmbH, Rheinstetten, Germany), ACD (ACD labs)	ACD (ACD labs), AMIX (BrukerBioSpin GmbH, Rheinstetten, Germany), vendor-specific software, Mnova	Automics (Softpedia), NMRFx, NMRPipe [88], BAYESIL [89], *R*-package AlpsNMR [90], *R*-package speaq [91]	ChromA [92], Chromaligner [93], MetAlign [94], MZmine [95,96], MZmine 2 [97], OpenMS [98], XCMS [99], XCMS^2^ [100], MAVEN [101], eRah [102]
metabolic feature extraction	extract signal intensities in untargeted manner from spectra to perform subsequent statistical analysis, reduce dimensionality, minimize effects from peak position variations across different spectra		equidistant bucketing/binning, Gaussian binning [103], adaptive binning [104], adaptive intelligent binning [105], dynamic adaptive binning [106], SRV [107], JBA [108], peak picking, manual/automatic definition of ROIs	equidistant bucketing/binning, peak detection/picking, manual/automatic definition of ROIs	AMIX (BrukerBioSpin GmbH, Rheinstetten, Germany), Chenomx (Chenomx Inc. Edmonton, Canada) [109]	vendor-specific software	*R*-package mQTL [110], *R*-package MWASTools [111], *R*-package speaq [91], *R*-package speaq 2.0 [112], *R*-package AlpsNMR [90]	MetaboAnalyst [113], MZmine [95,96], MZmine 2 [97], XCMS [99], MetAlign [94], MAVEN [101], MSClust [114], ROIMCR [115]
spectral deconvolution	deconvolute highly overlapping peak areas		curve fitting	MCR-ALS [116]	Chenomx (Chenomx Inc. Edmonton, Canada) [109]	vendor-specific software	BATMAN [117,118], decon1d [119], MetaboDecon1D [120], BAYESIL [89], non-linear peak fitting based on Voigt line shape model [121]	MetSign [122], DecoMetDIA [123], eRah [102]
missing value imputation	–	impute missing values to obtain full data matrix	–	half minimum imputation, mean value imputation, zero imputation, median value imputation, RF [124], MICE, kNN	–	vendor-specific software	–	MZmine [95,96], MetaboAnalyst [113], eRah [102], *R*-package mice [125], *R*-package VIM [126], *R*-package randomForest [127]
metabolite identification	identify metabolites in measured spectra		compare spectral features against reference spectra of pure compounds and/or query databases		Chenomx (Chenomx Inc. Edmonton, Canada) [109], AMIX (BrukerBioSpin GmbH, Rheinstetten, Germany) with BBIOREFCODE database, Aldrich FT-NMR (Sigma-Aldrich)	vendor-specific software	COLMAR [128], KnowItAll Metabolomics (BioRad Corp.), MetaboHunter [129], MetaboMiner [130], BAYESIL [89], ASICS [131], *R*-package speaq 2.0 [112]	MZmine 2 [97], OpenMS [98], XCMS [99], XCMS^2^ [100], MZedDB [132], eRah [102]
metabolite quantification	determine absolutely quantified concentrations of identified metabolites		accurately determine area under the curve of metabolite signal and reference with respect to known concentration of internal standard		Chenomx (Chenomx Inc. Edmonton, Canada) [109], AMIX (BrukerBioSpin GmbH, Rheinstetten, Germany)	vendor-specific software	BATMAN [117], [118], MetaboQuant [133], BAYESIL [89], AQuA [134], ASICS [131]	OpenMS [98]
metabolite data transformation	scaling of data in order to reduce data heteroscedasticity		e.g., log-transformation, variance stabilization transformation [135], auto-scaling, pareto scaling [136], mean centering		*R* Base, *R*-package vsn [137], *R*-package speaq 2.0 [112], Normalyzer [138], MetaPre [139]			
metabolite data normalization	minimize unwanted biological and/or technical variation between samples		e.g., creatinine normalization (for urine specimens), total spectral area normalization, normalization to internal standard, probabilistic quotient normalization [140], variance stabilization normalization [137], osmolality normalization, sample-specific normalization factors (e.g., volume), alternative: normalization-invariant zero-sum regression [77,78]		AMIX (BrukerBioSpin GmbH, Rheinstetten, Germany)	vendor-specific software	MetaboAnalyst [113], *R*-package AlpsNMR [90], *R*-package speaq 2.0 [112], Normalyzer [138], MetaPre [139], *R*-package zeroSum [77,78]	

**Table 3:** Overview of different statistical research goals and corresponding statistics/bioinformatics tools, including freely available R software packages, for metabolomics data analysis. Abbr.: ANOVA, analysis of variance; ASCA, analysis of variance—simultaneous component analysis; GGM, gaussian graphical model; LASSO, least absolute shrinkage and selection operator; MGM, mixed graphical model; OPLS-DA, orthogonal projections to latent structures—discriminant analysis; ORA, over-representation analysis; PH, proportional hazards; PLS-DA, partial least squares—discriminant analysis; WGCNA; weighted gene co-expression network analysis.

Research Goal	Example	LiteratureExample	Common Statistics/ Bioinformatics Method	Popular Statistics/ Bioinformatics Tools	*R* Software Packages	Further Reading
hypothesis testing	compare metabolite levels in CKD patients and healthy controls	[18]	hypothesis testing	Student’s *t*-test, ANOVA	>*R* Base: *t*.test, *R* Base: anova	[8,64]
multivariate bio-marker signature detection	multivariate metabolite signature to classify AKI vs. non-AKI patients	[19]	multivariate classification or linear regression	PLS-DA [148], OPLS-DA [149], support vector machine [150], Random Forest [124], LASSO regression [151], ridge regression [152], elastic net [153]	mixOmics [154], ropls [155], e1071 [156], randomForest [127], glmnet [157]	[8,64,158]
subgroup identification	exploratory identify CKD patient subgroups with different survival outcomes based on metabolic profiles	[32]	supervised/unsupervised machine learning	PCA [159], Hierarchical Clustering, Self-organizing maps [160]	*R* Base: prcomp, ropls [155], *R* Base: hclust, kohonen [161]	[8,64,160,162,163]
metabolome-wide association study	associations between all measured metabolites and eGFR, adjusted for age and sex	[35]	univariate/multivariate regression analysis (with confounder adjustment)	linear/logistic/Cox PH regression analysis	MWASTools [111]	[164]
statistical network analysis	exploratory identification of metabolite-metabolite associations	[30]	probabilistic graphical modeling, correlation networks	correlation network analysis, WGCNA [165], GGM [166], MGM [166]	corrr, WGCNA [167], GeneNet [168], mgm [169]	[166,170,171]
meta-analysis	combining *p*-values for creatinine and eGFR metabolite associations across multiple studies	[35]	regression model	fixed-effects model	metafor [172], meta [173]	[174]
time-to-event analysis	estimate the mortality of CKD patients based on a set of metabolites	[17]	survival analysis	Cox PH regression analysis [175], LASSO Cox PH regression [151], random survival forest [176]	survival [177], glmnet [157], randomForestSRC [176]	[177,178,179]
time-course analysis	analyze metabolite intensity changes over time under different CKD treatment conditions	[36]	time-course analysis	ASCA [180,181]	MetStaT [182], DESeq2 [183]	[184]
pathway (enrichment) analysis	identify set of metabolites differentiating non-CKD and CKD patients with affiliation to a specific pathway	[23]	hypergeometric test, regression model	MSEA [185], ORA, global test [186]	FELLA [187], Lilikoi [188], globaltest [186]	[171,189]
